# An update on lipid apheresis for familial hypercholesterolemia

**DOI:** 10.1007/s00467-022-05541-1

**Published:** 2022-04-25

**Authors:** Christina Taylan, Lutz T. Weber

**Affiliations:** Pediatric Nephrology, Faculty of Medicine, Children’s and Adolescents’ Hospital, University Hospital of Cologne, University of Cologne, Cologne, Germany

**Keywords:** Lipid apheresis, Pediatric therapeutic apheresis, Lipid-lowering treatment, Homozygous familial hypercholesterolemia

## Abstract

Familial hypercholesterolemia (FH) is an inherited metabolic defect leading to increased total cholesterol and low-density cholesterol (LDL) from birth onwards. Homozygous FH, presenting with clear clinical features, has a prevalence of ~ 1 per million. Prevalence of heterozygous FH is 1/500 European population. Atherosclerotic burden depends on the degree and duration of high LDL exposure. In severe cases, early detection is critical, and aggressive lipid-lowering therapies should begin in early childhood to reduce coronary heart disease risk. Pediatric therapeutic concepts correspond to adults and are orientated at LDL plasma concentration. Mean LDL plasma target value during treatment is < 135 mg/dL. Medication in childhood consists of ezetemibe, statins, resins, and PCSK-9 inhibitors, with consideration for age restrictions. Only a minority achieve the treatment target with drug therapy alone. Therapeutic apheresis for the treatment of hypercholesterolemia selectively removes lipoproteins from blood (lipid apheresis (LA)). LA has a long tradition in adult medicine and is also safely used in children by a variety of methods, if customized to special pediatric needs. LA reduces cholesterol levels independently of residual LDL-receptor function and not only achieves reduction or disappearance of xanthomas but also inhibits progression of or mitigates aortic valve stenosis and supravalvular aortic stenosis as well as coronary artery and other atherosclerotic lesions. Cardiovascular prognosis of patients with otherwise untreatable FH depends largely on timely use of LA. Taking into account LA as a lifelong treatment, starting early in childhood, it is important to accommodate therapy modalities, such as treatment frequency and point of time, into the life of the individual.

**Supplementary Information:**

The online version contains supplementary material available at 10.1007/s00467-022-05541-1.

## Introduction

Therapeutic apheresis (TA) was used back in 1952 for the first time to treat a patient with multiple myeloma and hyperviscosity. From the patient’s whole blood, erythrocytes were separated by gravity and given back to the patient. The plasma was disposed [1]. Since then, the method has been modified to be used for therapeutic plasma exchange from the early 1970s on [2]. Impressive changes in the safety and efficacy technology of the machines have now made routine TA procedures possible. Examples of such improvements comprise the following:

- Blood lines and filters that enable lower extracorporeal volumes with a reduced risk of hypovolemia and red blood cell (RBC) loss
- Machines are now able to detect blood clots and air in lines and measure dangerous access pressures.
- Anticoagulation regimens adapted to the needs of patients
- Decreased size and weight of the devices allowing greater mobility

A number of technically sophisticated TA machines are available today allowing a safe and easy performance of the procedure. The improvements made procedures faster, more convenient for the medical staff, and safer for patients [3]. Dependent on the machine used, blood tubing is possible with a volume of 70 mL, and the smallest blood separation filter has a volume of 25 mL. This extracorporeal volume has to be compared to the estimated total blood volume of a child, which is 65–85 mL/kg according to age. So, even small children can be treated safely when some precautions, like priming of the tubing with colloidal fluids and close monitoring of blood pressure and well-being of the patient, are taken. These developments and the increasing experience finally made it possible to use TA in children as well.

Since then, TA has become the established standard of therapy in the treatment of many diseases in the fields of pediatric neurology, nephrology, hemato-oncology, intensive care medicine, and gastroenterology. Throughout the last decade, TA has also been increasingly used as a treatment option in pediatric lipid disorders.

## Epidemiology of familial hypercholesterolemia

Familial hypercholesterolemia (FH) describes the condition of an inherited fat metabolism defect that leads to increased total cholesterol and low-density cholesterol (LDL) from birth on. Pathological forms can be present in homozygous, heterozygous, or compound-heterozygous forms. The resulting level of LDL is dependent on the underlying mutation. About one in one million people suffers from homozygous FH (HoFH) with LDL receptor (LDLR) mutation and clear clinical symptoms [4]. The prevalence of heterozygous FH (HeFH) patients with LDL receptor mutations is about one in 500 of the general European population [5], but these figures do not include populations with known higher prevalence throughout the world (e.g., Christian Lebanese in Lebanon, French-Canadian in Quebec, South African Afrikaner, Ashkenazi Jews) [6]. Based on the estimated prevalence of one in 500, up to 34 million individuals worldwide might suffer from FH. However, the prevalence of FH is unknown in 90% of the world’s countries, as only 17 out of 195 countries (9%) of the world have reported a FH prevalence for their general population [7]. Advances in genetic diagnostics technology are likely to diagnose more cases of definitive HoFH worldwide in the future.

A metanalysis by Beheshti et al. in 2020 analyzed the prevalence of FH in patients with ischemic heart disease (IHD), premature IHD, and severe hypercholesterolemia (≥ 190 mg/dL) in comparison with the general population [7]. IHD had 10 times higher frequency in patients with FH compared to subjects in the general population. Patients with premature IHD carried the underlying disease FH with 20 times the frequency, and patients with severe hypercholesterolemia had a 23-fold higher prevalence of FH.

## Genetic pathogenesis of familial hypercholesterolemia

**Fig. 1:**
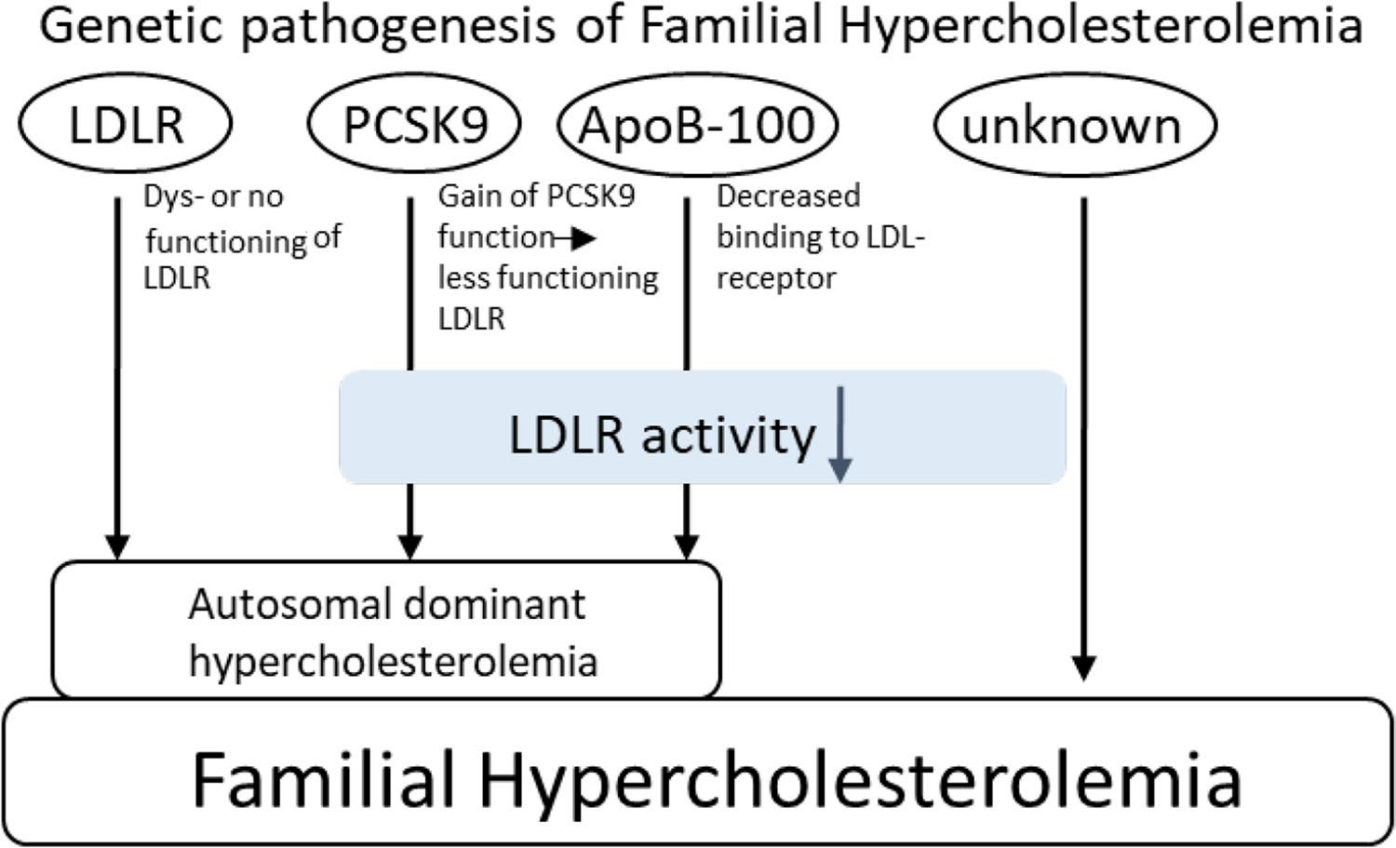
Genetic pathogenesis of familial hypercholesterolemia. LDLR: low-density lipoprotein receptor. PCSK9: Proprotein convertase subtilisin/kexin type 9. Apo B-100: Apolipoprotein B-100

FH can be caused by several genetic mutations (Fig. 1). More than 1400 point mutations and small deletions or insertions in the LDL-receptor (*LDLR*) gene are known in connection with FH. Among these 1400 variations of the *LDLR* gene, 58.5% were identified as missense mutations, 21.7% as small deletions or insertions, 10.4% as nonsense, and 9.4% as splice mutations [8]. All mutations can occur as hetero-, compound hetero-, or homozygous traits. The severity of hyperlipidemia and its drug response depend on the type of mutation. LDL receptor (LDLR) mutations resulting in a nonfunctioning LDL receptor (0 mutation) will be resistant to all medications that catalyze residual LDLRs to reduce serum LDL, such as β-hydroxy β-methylglutaryl-CoA (HMG-CoA) reductase inhibitors (statins) and proprotein convertase subtilisin/kexin type 9 (PCSK9) inhibitors. *LDLR* gene mutations are causative in 60–80% of genetically diagnosed FH [9].

Due to reduced function and number of LDLR, the uptake of LDL into the liver cell is disturbed, and subsequently, hepatic cholesterol synthesis is increased. While prevalence of LDL receptor mutations is reported to be high in white western populations, it is lower in other ethnic groups [10].

Some of the mutations are of special interest in the pediatric cohort, because of their early clinical onset:

- Patients with apolipoprotein-B (Apo B)-100 mutations have similar clinical manifestations as those with classical FH with *LDLR* mutations but show slightly lower LDL concentrations in general.
- A gain-of-function mutation in the *PCSK9* gene decreases LDL receptors and subsequently causes high LDL cholesterol [11].
- An autosomal recessive variant of high LDL cholesterol is caused by a mutation in the LDL receptor adapter protein 1 (LDLRAP1). LDLRAP1 contributes to the efficient endocytosis of LDL-LDLR complexes. LDLRAP1 is involved in the endocytosis of the LDL receptor. Patients with LDLRAP1 mutations present with huge xanthomas associated with high LDL [12].

## Clinical consequences and therapeutic options

All patients suffering from one of the abovementioned mutations display findings like very high LDL plasma concentrations and fat deposits in typical locations, e.g., in the tendons (xanthomas) and around the eyes (xanthelasma). Elevated LDL is held responsible for vascular disease with the risk of premature coronary heart disease (CHD) [13]. The risk of developing atherosclerosis depends on the level of LDL plasma concentration and the duration an organism is exposed to these levels. So, vascular disease becomes manifest with variable symptoms up to aortic stenosis due to calcification [14]. Early detection is extremely important in severe cases, and consistent lipid-lowering therapies should begin in early childhood to reduce the risk of CHD [15, 16]. In general, clinical consequences of CHD do not manifest until adulthood, but children with severe FH can already present signs of early atherosclerosis development, namely endothelial dysfunction and thickening of the arterial vessel wall [17]. Currently, statins are part of almost every drug treatment regimen for hypercholesterolemia. They are often combined with cholesterol uptake inhibitors and cholesterol elimination accelerators. LDL is bound from the extracellular space to LDLR on the liver and other cell membranes, thus taking care of the transport of LDL into the cells. Statins catalyze the uptake of LDL by upregulating LDLR. Hence, efficacy of statins relies on residual function of LDLR. Therefore, they display a high inter-individual variance in efficacy. In patients with an *LDLR* gene mutation that results in a complete lack of LDLR or complete receptor dysfunction, statins have no effect at all [18].

**Fig. 2:**
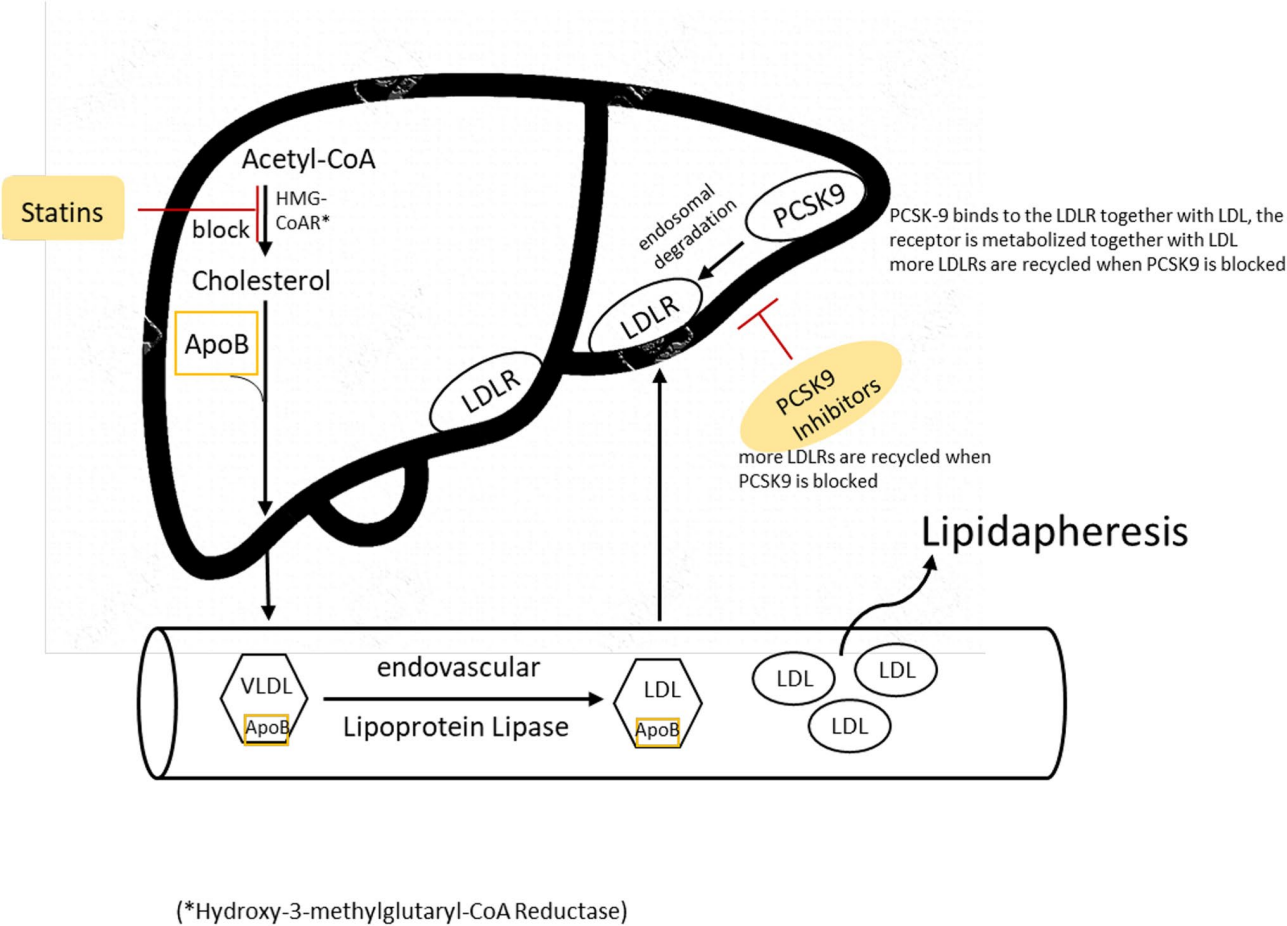
Lipid metabolism and therapeutic aims. Acetyl-CoA: Acetyl Coenzyme A Carboxylase LDLR: low-density lipoprotein receptor. PCSK9: Proprotein convertase subtilisin/kexin type 9. ApoB: Apolipoprotein B-100. VLDL: very low-density lipoprotein. LDL: low-density lipoprotein

PCSK-9 binds to the LDLR together with LDL. The binding of PCSK-9 inhibits LDRL recycling, and the receptor is metabolized together with LDL (Fig. 2). More LDLRs are recycled when PCSK9 is blocked, so more LDLRs are present on the surface of cells, and more LDL particles can be removed from the extracellular fluid [19]. In this way, blocking PCSK9 lowers LDL levels in the blood [20], unless there are none or no functioning LDLRs due to the underlying genetic mutation [18].

The curative treatment approach to FH is liver transplantation. There are some reports of successful transplantation and prevention of long-term vascular damage [21–23]. However, transplantation may not be considered the method of choice. The overall risk of organ transplantation, which is composed of surgical, immunological [24], and infectious [25] complications as well as side effects of lifelong immunosuppressive therapy [26], has to be weighed against those of LA, being a highly efficient treatment method that is usually very well tolerated. Timing of transplantation is an important issue, too, since vascular damage may not be reversible and an additional risk factor for transplantation-associated complications [26].

The goal of medical treatment of children with FH should be to lower the baseline LDL plasma level by 50%. Should the lipid-lowering drug combination of statins, ezetimibe, bile acid resins, and monoclonal antibodies against PSKK-9 not be sufficient to achieve this target, LA can be applied as an adjunctive treatment to remove LDL in patients with severe dyslipidemia.

Of note, lifestyle and diet changes are recommended at the beginning of any therapy. In hypercholesterolemia, a limited intake of saturated and trans-isomeric fatty acids (together up to approximately 8–12% of the energy intake) with replacement by monounsaturated fats (> 10% of energy) and moderate intake of polyunsaturated fatty acids (approximately 7–10% of energy), as well as limited cholesterol intake (up to approximately 200 mg/day in childhood or 250 mg/day in adolescents), are advised [27].

Certain risks, such as smoking and obesity, must be avoided at all costs. An additional risk for the premature development of cardiovascular events is the development of secondary diseases such as hypertension and diabetes mellitus. Nevertheless, it must be noted that even a strict adherence to all dietary requirements in genetically determined FH can only have little effect on serum LDL levels.

## Lipid apheresis

**Fig. 3:**
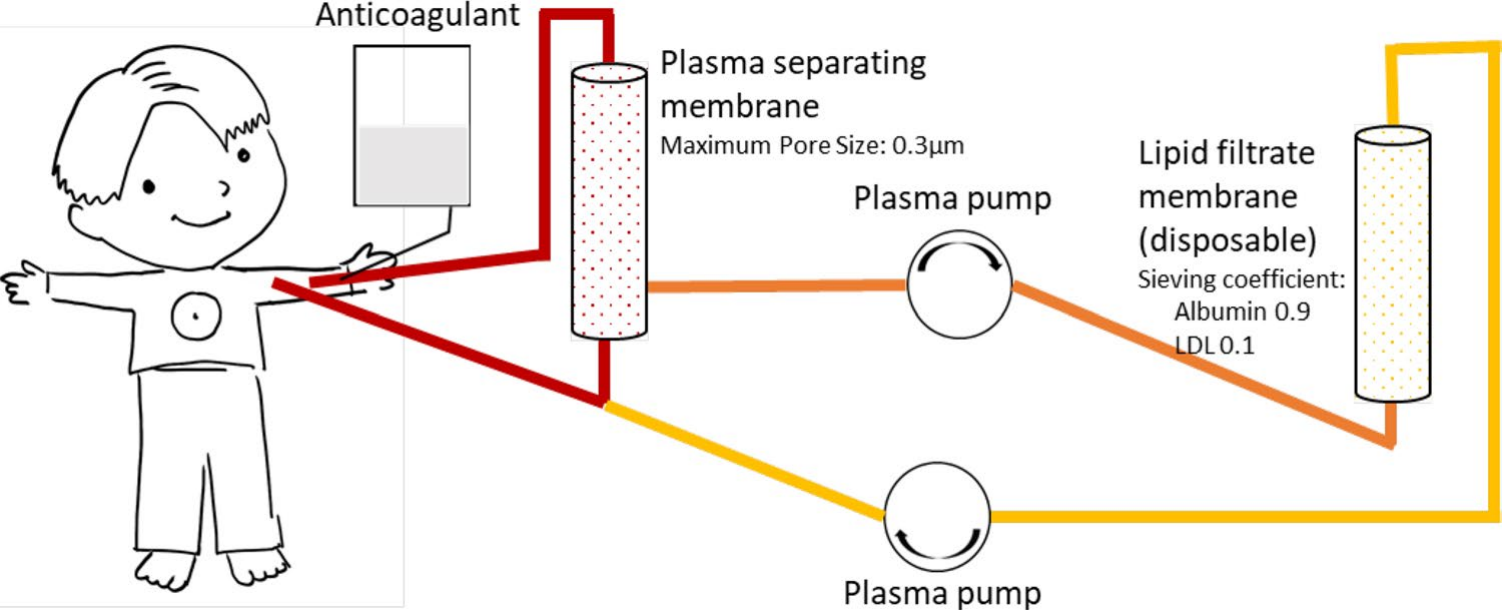
Schematic illustration of double filtration plasmapheresis

In 1980, the first report on long-term plasmapheresis for the treatment of hypercholesterolemia in two children was published by King and colleagues [21]. Since then, the safety aspect in particular has improved significantly, taking into account very small treatment volumes in pediatrics. Today, there are five basic methods of selectively removing lipoproteins from blood; all systems have shown a comparable ability to lower LDL cholesterol [22]. Figure 3 illustrates in detail the method which is currently used in most pediatric dialysis centers. The interested reader will find the illustrations for four more methods in the Supplementary Figs. 1–4.

### Double filtration

In this procedure, two filtration membranes connected in series with different pore sizes are used. Double filtration plasmapheresis (DFPP) is also known as membrane differential filtration, cascade filtration, or lipid filtration. The first filter separates the plasma from whole blood. The second filter separates plasma macromolecules such as lipoproteins based on their molecular weight and size. Due to the pore size limit of about 1,000,000 Da, the second membrane retains LDL particles with a molecular weight of about 2,300,000 Da. Smaller molecules can pass through the membrane and are not filtered. Although the reduction of LDL and lipoprotein(a) (Lp(a)) is similar to other methods, DFPP is less selective. Other relatively large plasma components will be reduced more drastically (e.g., HDL (35–50%), IgM (35%), and fibrinogen (50%)). In addition, the pores of the secondary membrane tend to clog, limiting the volume of plasma that can be treated. With time, the filtration properties have been improved by technological advances, and the newer cascade flow system (Asahi©, Japan) shows a good efficacy and safety profile in reducing LDL and Lp(a) while preserving HDL and immunoglobulins. This procedure is currently used in most German children's dialysis centers for LA.

### LDL adsorption due to binding based on immunoaffinity

The LDL adsorption method with the liposorber (Kaneka©) makes use of the selective ability of LDL to bind to dextran sulfate. After the blood plasma has been separated from the blood cells, the plasma is passed over columns containing the negatively charged ligand dextran sulfate in porous beads. The lipoproteins very-low-density lipoprotein (VLDL), LDL, and LP(a) containing the positively charged primary Apo B are selectively removed. Two small volume columns (LA15, 150 mL) are used. LDL is adsorbed in the columns and then eluted by flushing the column with high concentration sodium chloride (NaCl) (5%), thereby regenerating the column. The negatively charged LDL-adsorbing columns activate the blood coagulation system and consequently lead to an increase in bradykinin levels. Since one effect of angiotensin-converting enzyme (ACE) inhibitors is accelerated bradykinin degradation, combination therapy with ACE inhibitors and liposorber LA can cause anaphylactic symptoms and is contraindicated.

### LDL adsorption due to binding based on immunoaffinity

Another system using the immunoaffinity of LDL is the TheraSorb™-LDL system (Miltenyi Biotec©). It also first separates the plasma and then binds LDL and Lp(a) particles using a column filled with sepharose beads that are covalently attached to antihuman sheep Apo B antibodies. After the plasma has been separated, it is directed into the adsorption column where LDL, VLDL, and Lp(a) bind to the beads. The plasma cleaned in this way is returned to the patient together with the cellular blood components. The TheraSorb™ LDL is a two-column system in which adsorption and regeneration take place alternately and allow the treatment of theoretically infinitely large plasma volumes without saturation. This immunoadsorption (IA) system is the most selective of the various LA technologies but harbors the inherent risk of leaching of the sorbent into the recirculated plasma, which can produce febrile or anaphylactic reactions. Here, too, the use of ACE inhibitors is contraindicated.

### Heparin-induced precipitation

In the heparin-induced extracorporeal LDL-precipitation (H.E.L.P. by Braun©) process, the first step is to separate the plasma from the other blood components. Then, heparin and an acetate buffer are added, lowering the pH of the plasma. This changes the surface charge of LDL and Lp(a), which leads to increased binding to the heparin used. The resulting precipitates are filtered and then removed from the plasma circuit. The excess heparin is eliminated by adsorption. The physiological pH value is then restored by removing the buffer solution by means of dialysis. The purified blood is finally returned to the patient at the end of the treatment. There is no contraindication to ACE inhibitors.

### DALI (direct adsorption of lipoproteins)

The central element of DALI® LA (Fresenius©) is the adsorber column which also uses the immunoaffinity of LDL. In the DALI® adsorber, the positively charged Apo B-100 regions of LDL, VLDL, and Lp(a) particles selectively bind to negatively charged polyacrylamide immobilized on Eupergit® beads. The DALI adsorber is available in different sizes (DALI® 500, DALI® 750) which allows individual adjustment to the patient. LDL can be reduced by 64 to 76% during treatment. Negatively charged ligands in the adsorber lead to the activation of bradykinin, which is rapidly broken down by the angiotensin-converting enzyme (ACE). Therefore, angiotensin-converting enzyme inhibitors are also contraindicated in DALI lipid apheresis.

## Lipid apheresis in children

The homozygous or compound heterozygous (c-hetFH) forms of FH are the main indication for the use of LA in children and adolescents. Here, the mutation causes a full defect in the particular aspect of the lipid metabolism, leaving the affected individual with very high LDL levels. In addition to drug therapy, regular LA is a component of therapy. Especially, the more effective drugs, such as statins and PCSK9 inhibitors, utilize the few existing or less-functioning LDL receptors for their mechanism of action. Therefore, their approach requires a residual function of the LDL receptor and cannot work when the LDL receptor is completely defective.

The LDL mean target value during treatment is < 135 mg/dL. Only a minority of the children affected can achieve this target with drug therapy alone [18]. Delays in diagnosis, lack of monitoring, and treatment are some of the underlying reasons [23]. LA lowers cholesterol levels independently of the residual activity of the LDL receptors. In this way, a reduction or disappearance of xanthoma can be achieved, and, even more importantly, progression of vascular disease can be prevented, or even its improvement is possible [24, 25]. Additional benefits of LA beyond lowering LDL cholesterol include positive effects on erythrocyte and platelet membranes, rheology, and vascular endothelium (i.e., induction of vasodilator factors, cytokines, and anti-inflammatory effects) [26]. Atherosclerosis is characterized by a multifocal vascular inflammatory process, and a large number of inflammatory biomarkers have been identified in the last decade [27], which can be filtered by LA. The proteins bound in the columns were analyzed and showed a column-type dependent protein loss. The highest number of protein spots was found in patients treated with DALI, followed by HELP and DFPP. These proteins are involved in the coagulation pathway (e.g., kininogen1) and have adhesive (e.g., fibronectin), rheological (e.g., fibrinogen), and immunological/inflammatory properties (e.g., complement components) [28]. Eventually, the receptor — and flow-mediated endothelial cell-dependent vasodilatory capacity — is inhibited by inactivation of nitric oxide (NO) by oxidized LDL, decreased receptor-mediated NO release, and reduced expression of endothelial NO synthase [29] and may be restored by LA.

LA inhibits the expression of cell adhesion molecules (ICAM-1, ELAM-1, etc.) and thrombus formation by reducing fibrinogen and coagulation factors. After LA, LDL cannot be easily oxidized, and LA has an additional anti-atherosclerotic effect by enhancing LDL subtypes [30]. By this, LA has a further protective effect via modulated rheology.

**Fig. 4:**
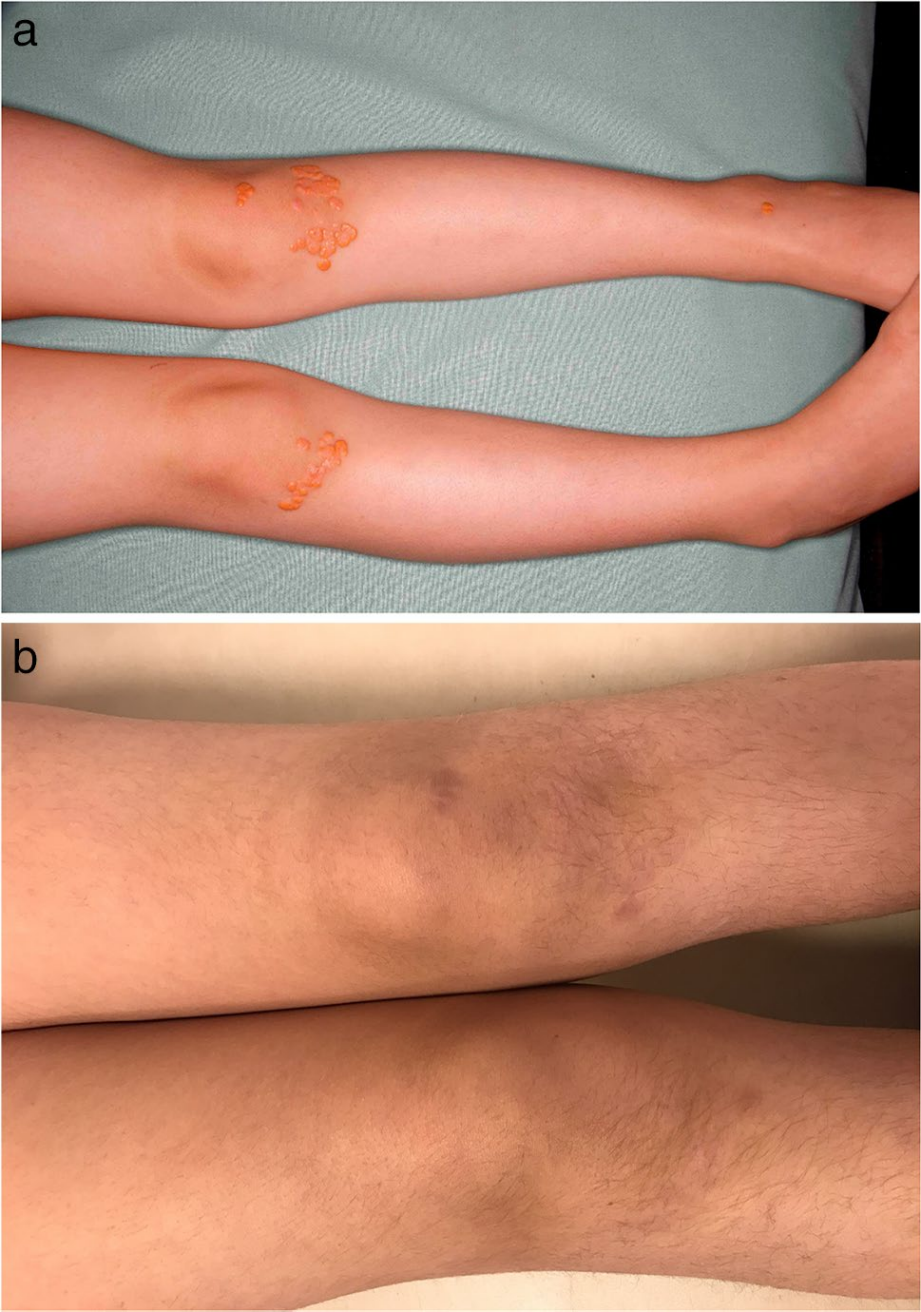
**a** and **b**: Lipid skin deposits (knees) before start of lipid apheresis (**a**) [32] and lipid skin deposits (knees) after 12 months of lipid apheresis twice a week (**b**)

**Fig. 5:**
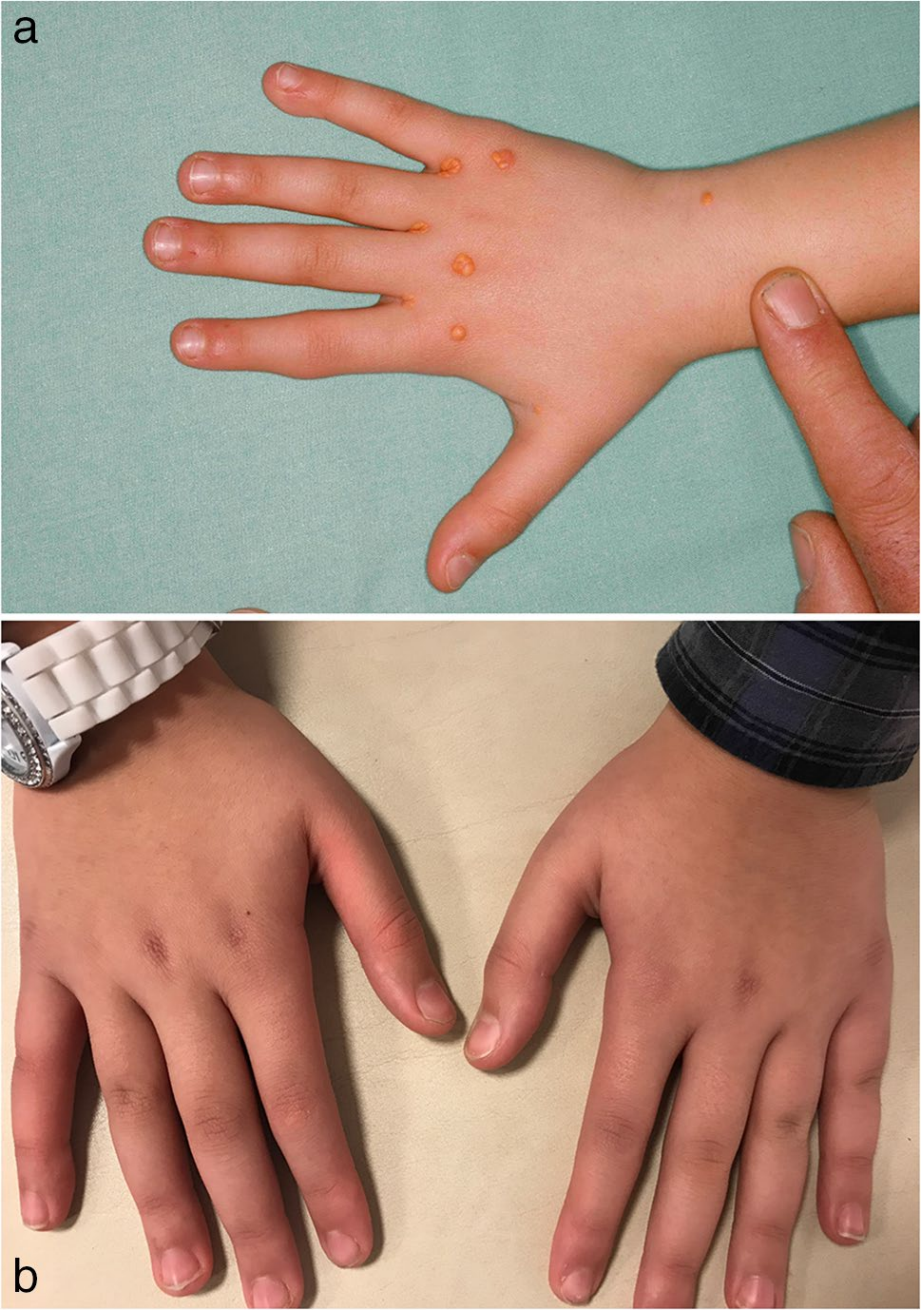
**a** and **b** Lipid skin deposits (right hand) before the start of lipid apheresis (**a**) [32] and lipid skin deposits (hands) after 12 months of lipid apheresis twice a week (**b**)

Nevertheless, high mortality in the affected cohort of patients remains an unsolved problem, and it is crucial to identify additional risk factors, such as elevated body mass index, kidney disease, liver disease, hypertension, and coagulopathies for proper treatment of these patients. In 2018, Klaus et al. retrospectively analyzed 17 patients with biallelic FH commencing chronic LA before the age of 18 years. With a combined therapy with drugs and LA, only three of 17 patients were able to achieve a mean LDL plasma concentration below the pediatric target value of 135 mg/dL. In only two of 17 patients, the frequency of LA was twice a week, and notably, these two children reached target values [31]. Increasing the LA frequency to twice a week can further reduce mean LDL and has been shown to be more effective when compared to increasing treated plasma volume [32]. Since increasing the frequency can avoid a premature rebound of the LDL, it is not necessary to treat high plasma volumes per session. Instead, it is sufficient to treat the simple to 1.5-fold plasma volume. In 2020, we analyzed the same patient cohort (plus 7 new patients, resulting in a total of 24 patients) again and found that in the interim, 11 of these patients achieved mean LDL concentrations below the pediatric target value of 135 mg/dL. All of these 11 patients had been treated with LA twice a week [18]. This intensive form of therapy was well tolerated by the children and did not cause much absence from school, since we were able to treat the children with an individual schedule that fitted social life. Skin lipid deposits, such as xanthomas and xanthelasmas, disappeared within months [32]. Regress of skin deposits is displayed in Figs. 4–5.

Early treatment of pediatric hyperlipidemia could be made possible through intensive family counseling and screening. International literature advises screening for FH starting at the age of 2 years, when one of the following events happen:

- A family member presents with FH.
- A plasma cholesterol level of ≥ 310 mg/dL in an adult or of ≥ 230 mg/dL in a child is measured.
- Presentation with premature CHD, tendon xanthomas, or sudden premature cardiac death [33].

The German guidelines recommend checking plasma lipid levels if there is any anamnestic evidence of hereditary dyslipidemia. This is defined as early cardiovascular disease in 1st- and 2nd-degree relatives before the age of 55 (men) or 60 years (women). Regardless of the family history, each child or adolescent should have one serum cholesterol level performed (total cholesterol) as part of a preventive medical checkup, preferably in preschool age [34]. The diagnosis of children with dyslipidemia, causing an increased risk of premature atherosclerosis, requires a thorough investigation of secondary causes by taking into account anamnesis, clinical examination, and, if necessary, targeted laboratory diagnostics (e.g., TSH (thyroid-stimulating hormone), serum creatinine, alanine aminotransferase, aspartate aminotransferase, γGT (γ-glutamyltransferase), bilirubin, fasting blood glucose). Lipid levels vary with age, especially during puberty.

Wiegman et al. were able to show that in the Netherlands, in children aged under 18 years, a LDL plasma concentration of > 150 mg/dL predicted the carrier status of an LDLR mutation with a post-test probability of 0.98 [35].

Once a parent has been diagnosed clinically or in laboratory tests, genetic testing should be done in that patient first, and if the diagnosis is genetically confirmed, genetic testing should also be done in their children with clinical abnormalities. Genetic testing is the gold standard for diagnosing the condition. A pathogenic mutation in one of the LDLR, Apo B-100, and PCSK9 genes can be identified in 70–80% of FH [9]. Even cases without a known mutation can represent a diagnosis of FH, especially in cases with a strong phenotype. Confirming the diagnosis also has an impact on the prognosis; a positive result from genetic testing is associated with prognosis of higher risk for atherosclerotic cardiovascular disease (ASCVD) than for those who do not have a mutation [36]. Target LDL levels in children are internationally discussed; to date, expert consensus-recommended targets range from < 100 mg/dL to < 160 mg/dL [37, 38]; in Germany, LDL plasma target is < 135 mg/dL, and an effective treatment should result in a reduction of 50% from pre-treatment levels in children aged 8–10 years [39]. Lipid-lowering therapy should start as early in life as possible. Ezetemibe (approved in Germany from the age of 6 years), statins (e.g., rosuvastatin approved in Germany from the age of 6 years), resins (approved in Germany for use in children with no specific age statement), and PCSK-9 inhibitors (evolocumab approved in Germany from the age of 10 years) give the opportunity to start medication from a relatively early age.

In cases with a very early onset of clinical features of premature cardiovascular disease, LA should be initiated from as young an age as possible. The benefit of an effective treatment and the risk of a permanent central catheter, when the child is too small for an arteriovenous (AV) fistula, must be considered and discussed. There is currently no differentiation between children and adults as for the indication as well as absolute treatment targets of LA, due to the apheresis committee of the medical associations. In the future, absolute treatment targets should be adapted to patient age and clinical condition and should therefore be as low as possible.

In 2013, Nordestgaard et al. published recommended lipid target values for patients with FH. For children, an LDL cholesterol target of < 135 mg/dL has been advised, whereas in adults, target levels were stricter and risk stratified. Adults should not exceed an LDL > 100 mg/dL, and adults with known CHD or diabetes should have LDL values < 70 mg/dL [40]. This was changed in 2019 when the guidelines of the European Society of Cardiology (ESC) and the European Atherosclerosis Society (EAS) were published. The main innovation was a significant reduction in the target values for LDL-C (55 mg/dL) for adults. Target values for children have not been changed. The German Cardiac Society essentially adopted this European guideline of the ESC/EAS as a German guideline as well as the German Lipid Liga. The most recent version originates from 2019 to 2020 [41].

## Specific technical requirements in children

If safe vascular access can be established, the use of apheresis in children is possible regardless of the patient’s body weight. Of note, LA has no negative effects on development or growth [24]. Since it is a lifelong therapy, special care must be taken to ensure that the children are given maximum support in their physical, mental, and cognitive development, and that the treatment is designed to be as stress-free as possible. Time-consuming or uncomfortable examinations, such as instrumental or laboratory tests, should be carried out during the apheresis treatment. The same requirements are placed on a children’s apheresis center as are on a children’s dialysis center. A psychosocial team is just as necessary as school support and the possibility of nutritional advice.

Technically, it is important to adapt apheresis procedures in young children to the patient’s height and weight, since apheresis devices and the controlling software are usually designed for use in adults. Particular attention should be paid to the points outlined below.

### Vascular access

In most pediatric patients, the native veins are not suitable for performing apheresis procedures using a peripheral venous line. A blood flow of at least 80 mL/min must be achievable. Therefore, in the majority of children who require long-term apheresis, an AV fistula is created, or if this is not possible, a double-lumen central venous catheter is inserted. The length and diameter of the catheter must be selected depending on the size of the child and the quality of the child’s veins. When taking the high risk of catheter-related sepsis of long-term therapy into account, wherever possible, the creation of an AV fistula is a preferred option. A surgical requirement for the creation of a shunt is an artery of at least 1-mm diameter. In addition, the child should be cooperative enough to tolerate the two-needle system during an approximately 2-h procedure. Fistula thrombosis has been observed more often in hypercholesterolemia patients than in dialysis patients in our institution (unpublished data), maybe due to even worse vessel quality and more thrombogenic material in these patients. In 2017, the US Food and Drug Administration approved an intravascular port device specifically designed for apheresis therapy, which can be used alternatively [42–45].

### Extracorporeal volume and blood priming

The extracorporeal volume (ECV) of the device should be taken into account when planning a LA procedure in a small child. The ECV depends on the device used and the associated line system. It varies between 95 and 400 ml. While adult patients can easily tolerate the transient volume loss, smaller pediatric patients are at risk of complications. A drop in blood pressure, dizziness, or vomiting may be signs of hypovolemia. If the ECV of the device exceeds 10–15% of the child’s blood volume, it may be necessary to transfuse blood at the beginning of treatment. Priming with human albumin or 0.9% NaCl is also possible. The ECV is clearly defined for all apheresis devices/procedures; the total blood volume of the patient must always be calculated. Total blood volume in children is estimated at 65–85 mL/kg depending on age. Apheresis procedures can be performed in young children without altering the patient’s blood volume or red blood cell mass during the procedure, given the unique needs of the pediatric cohort. Vice versa, a very high hematocrit > 35% may harbor the risk of clotting; therefore, dilution can be taken into account.

### Anticoagulation

As with other extracorporeal procedures, apheresis requires anticoagulation to prevent clotting in the device and extracorporeal circuit. This can be achieved with sodium citrate, which prevents clotting by chelating calcium. The coagulation system of the blood is calcium-dependent; a lowering of the calcium level leads to an inhibition of activation. A major advantage of citrate anticoagulation is its exclusive effect in the extracorporeal circuit; citrate allows the system to be anticoagulated without the risk of bleeding associated with systemic anticoagulation. Risk of anticoagulation with citrate is transient hypocalcemia.

Heparin can also be used for anticoagulation for apheresis procedures. Here, the administration of heparin leads to systemic and extracorporeal anticoagulation. Patients who receive heparin have a slightly increased risk for bleeding during and immediately after the procedure. None of the procedures is clearly superior to another.

## Summary and conclusion

LA is a safe treatment method with a long tradition in adult medicine. Thanks to the development over the last 40 years, it can also be safely used in children, if it is customized to some specific pediatric needs. There are different methods of application that are still evolving. The prognosis of the cardiovascular health of patients with otherwise untreatable FH depends largely on the timely use of LA. Taking into account that LA is a lifelong and time-consuming treatment, starting early in childhood, it is very important to fit the therapy modalities, such as treatment frequency and point of time, into the life of the individual. For example, emphasis should be laid on enabling school attendance as well as leisure activities. Both the affected child and their family need support to cope with the burden of a chronic life-threatening disease requiring lifelong therapy. Therefore, children should always be treated in specialized pediatric apheresis departments in which social care, psychological support, and school coaching can be provided.

## Key summary points

- LA is a well-established blood purification procedure that is safe to use in children.
- Effective treatment of FH should be started as early as possible in life.
- The choice of drug treatment as well as the intensity and frequency of LA depend on the underlying mutation and must be modified until an optimal result has been achieved.
- Due to the very time-consuming therapy, it must be chosen in a way that creates as little stress as possible for the patient, and psychosocial support must be offered.

## Multiple-choice questions

1. Which of the statements below is wrong?

- It is recommended to check plasma lipid levels in children if there is a 1st and 2nd degree relative before the age of 55 (men) or 60 years (women) with early cardiovascular disease.
- An affected parent should undergo genetic testing first and when the diagnosis has been confirmed, genetic testing in the potentially affected child should be performed.
- It is recommended to check plasma lipid levels in every child once, regardless of anamnestic hints or clinical features.
- It is only recommended to look for secondary reasons for elevated lipid levels if no pathogenic mutation can be found.

2) Which of the statements below is correct?

- Serum lipid levels are age dependent.
- LDL-target levels are the same in children and adults.
- LDL-target levels can only be reached with a multimodal treatment of medication and lipid apheresis.
- LDL-target levels can only be reached with a multimodal treatment of medication and lipid apheresis twice a week.

3) Which of the statements below is wrong?

- A mutation in the *PCSK9* gene decreases LDL receptors and subsequently causes hypercholesterolemia.
- LDL receptor mutations can result in medication resistance.
- *LDLR* gene mutations are causative in 60–80% of genetically diagnosed cases of hypercholesteronemia.
- If an underlying mutation can be identified, the treatment should always be a multimodal intense therapy because all mutations cause LDL plasma values elevated to a comparable level.

4) Which is the correct next step in diagnostics or therapy when a child . . .

- is newly diagnosed and has no medication yet? Start with a combination of statins, intestinal cholesterol uptake inhibitors and PCSK9 inhibitors to lower the burden of LDL as fast as possible.
- is on statins, intestinal cholesterol uptake inhibitors and PCSK9 inhibitors, and does not show a satisfactory decrease in LDL levels? Check underlying mutation and eventually terminate statins and PCSK9 inhibitors.
- is on lipid apheresis and does not show a satisfactory decrease in LDL levels? Increase the plasma volume to treat, because more treated plasma in a session results in a long-lasting decrease in mean LDL.
- is on lipid apheresis and does not show a satisfactory decrease in LDL levels? Check eluted liquids for co-eliminated factors, because they can be washed out completely.

5) Which of the statements below is correct?

- Endothelial dysfunction and thickening of the arterial vessel wall in hypercholesterolemia can already be present in children.
- Severe atherosclerotic lesions like aortic valve stenosis and supravalvular aortic stenosis cannot be present before adolescence.
- Lipid apheresis does not influence coagulation and rheological pathways, adhesive proteins, immunological/inflammatory properties and vessel compliance.
- Other substances besides LDL can only be found in the DALI column.

## Supplementary Information

Below is the link to the electronic supplementary material.

Supplementary file1 (DOCX 383 KB)
