# Supplementary material for: An update on lipid apheresis for familial hypercholesterolemia
Source: Pediatr Nephrol. 2022 Apr 25;38(2):371–82. doi: 10.1007/s00467-022-05541-1 (PMC9763149; doi:10.1007/s00467-022-05541-1)
Supplement: Supplementary file 1 — Supplementary file1 (DOCX 383 KB) [file 467_2022_5541_MOESM1_ESM.docx]

**Supplemetary material for**

**“An update on lipidapheresis for familial hypercholesterolemia”**

**In**

**“Pediatric Nephrology”**

**by**

**Christina Taylan^1^ und Lutz T Weber^1^**

**^1^ Pediatric Nephrology, Children’s and Adolescents’ Hospital, University Hospital of Cologne, Faculty of Medicine, University of Cologne, Germany**

**Correspondency:**

**Dr. med. Christina Taylan**

**Klinik und Poliklinik für Kinder- und Jugendmedizin, Uniklinik Köln**

**Pädiatrische Nephrologie**

**Kerpener Str. 62**

**50937 Köln**

[**christina.taylan@uk-koeln.de**](mailto:christina.taylan@uk-koeln.de)

**Fig. 1**

**LDL Adsorption due to binding based on immunoaffinity**

**
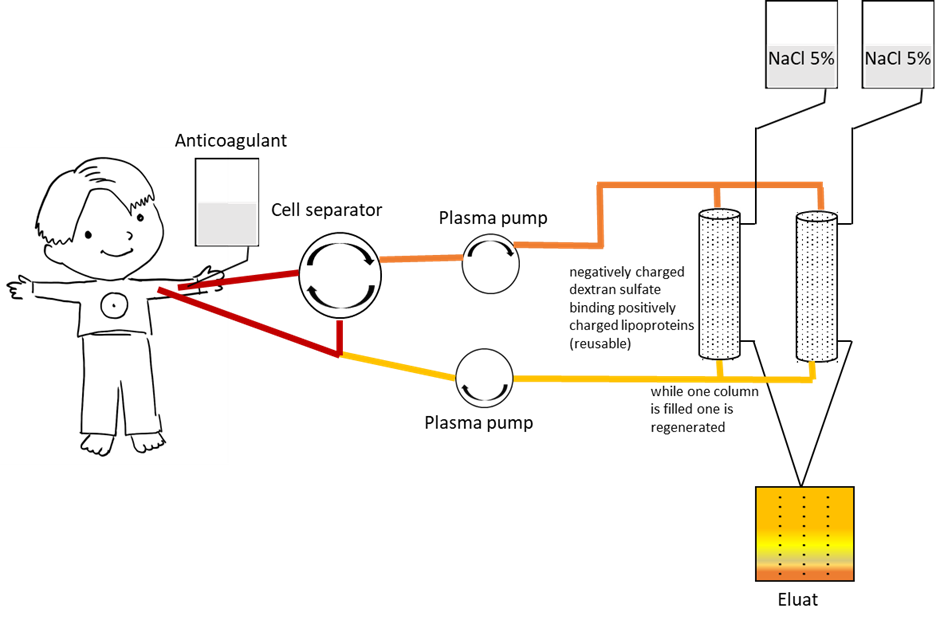
**

Fig. 1: Schematic illustration of LDL Dextran Sulfate Adsorption

**Fig. 2**

**LDL Adsorption due to binding based on immunoaffinity**

**
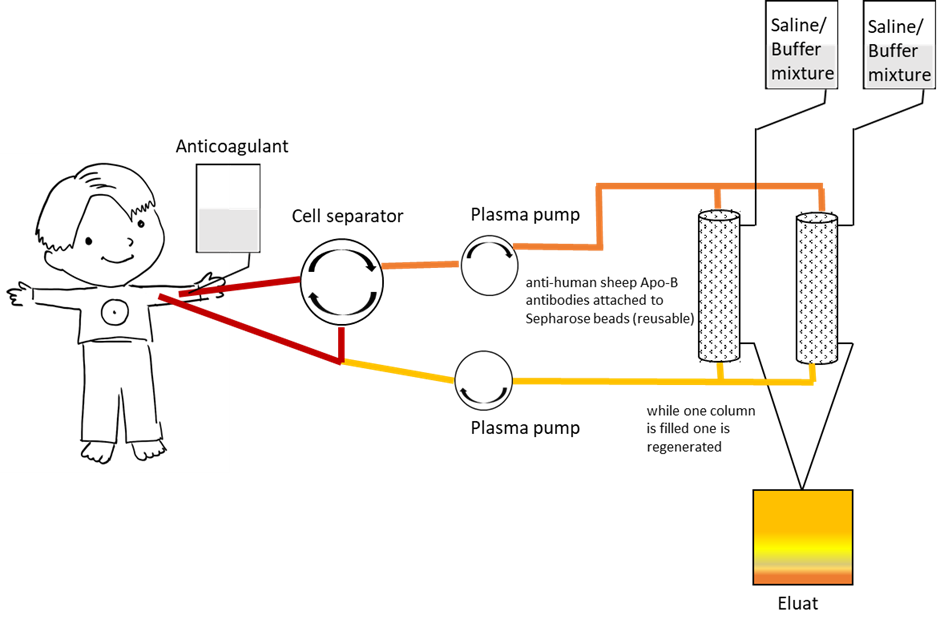
**

Fig. 2: Schematic illustration of LDL Sepharose beads Adsorption

**Fig. 3**

**Heparin-induced precipitation**

**
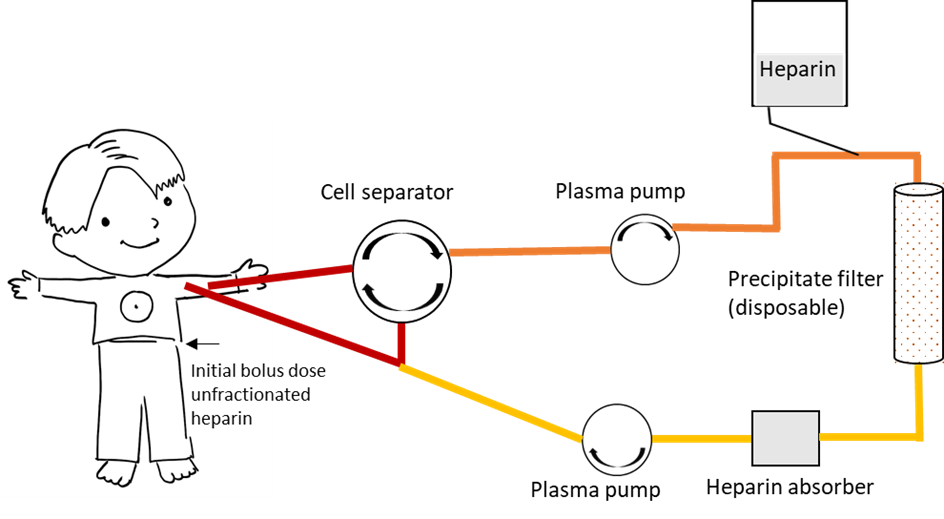
**

Fig. 3: Schematic illustration of LDL Heparin-induced precipitation

**Fig. 4**

**DALI (Direct Adsorption of Lipoproteins)**

**
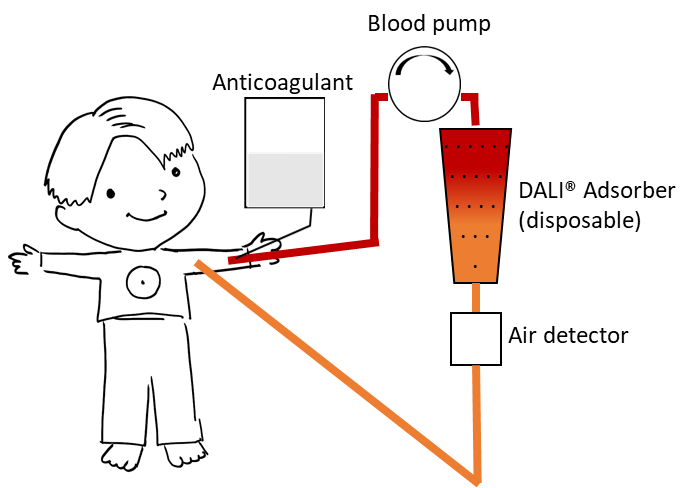
**

Fig. 4: Schematic illustration of Direct Adsorption of Lipoproteins
